# Evidence for Differential Assortative Female Preference in Association with Refugial Isolation of Rainbow Skinks in Australia's Tropical Rainforests

**DOI:** 10.1371/journal.pone.0003499

**Published:** 2008-10-29

**Authors:** Gaynor Dolman

**Affiliations:** 1 Australian National Wildlife Collection, CSIRO Sustainable Ecosystems, Canberra, Australia; 2 School of Integrative Biology, University of Queensland, St. Lucia, Australia; University of Otago, New Zealand

## Abstract

**Background:**

Divergence driven by female preference can give rise to pre-mating isolation more rapidly than post-mating isolation can evolve through the accumulation of allelic incompatibilities. Moreover pre-mating isolation may be more effective at maintaining morphological differentiation between divergent populations. In the context of Australian rainforest endemic skinks that were historically subjected to refugial isolation, this study examined the following predictions: 1) that assortative female preference is associated with more recent divergence of southern *C. rubrigularis* (S-RED) and *C. rhomboidalis* (BLUE), but not with deeply divergent S-RED and northern *C. rubrigularis* (N-RED); and 2) that upon secondary contact, morphological differentiation is maintained between S-RED and BLUE, whereas N-RED and S-RED remain morphogically indistinguishable.

**Principal Findings:**

Female preference trials found no evidence for assortative female preference between N-RED and S-RED, supporting a previous genetic hybrid zone study which inferred post-mating but no pre-mating isolation. In contrast there is evidence for assortative female preference between S-RED and BLUE, with BLUE females preferring to associate with BLUE males, but S-RED females showing no preference. Multi-locus coalescent analyses, used to estimate post-divergence gene-flow between proximally located S-RED and BLUE populations, rejected zero gene-flow from BLUE to S-RED and thus RED and BLUE have maintained morphological differentiation despite secondary contact. Morphometric analyses confirmed a lack of morphological divergence between N-RED and S-RED and established that BLUE is morphologically divergent from RED in traits other than throat colour.

**Conclusions/Significance:**

Long-term isolation has been sufficient to generate post-mating isolation but no morphological divergence between N-RED and S-RED. In contrast, greater morphological differentiation is associated with evidence for assortative female preference between more recently diverged S-RED and BLUE. Combined with previous estimates of lineage-wide gene flow, these results are consistent with the suggestion that assortative female preference is more effective than post-mating isolation in maintaining morphological differentiation between divergent populations.

## Introduction

While populations are isolated in separate refugia, they diverge through genetic drift and/or selection [1], but to maintain distinctiveness upon secondary contact, they must evolve substantial pre-mating or post-mating isolation. Post-mating isolation occurs when isolated populations accumulate allelic incompatibilities over time [2]–[4]. Both pre-mating and post-mating isolation between allopatric populations increase with genetic distance [5]–[7]. However unlike post-mating isolation, pre-mating isolation can evolve rapidly, through the action of sexual selection [8], [9]. This rapid evolution of pre-mating isolation is often manifested as divergence in sexually dimorphic characters [10]–[12]. Pre-mating isolation is often regarded as a more important or more effective barrier to gene flow than post-zygotic isolation or post-mating pre-zygotic isolation [13], [14] and thus morphological differentiation of divergent populations should be more readily maintained upon secondary contact.

The origin of high species diversity in tropical rainforests is a subject of ongoing investigation. While the role of refugial isolation remains speculative for most rainforest systems, the contraction of Australia's tropical rainforests into isolated refugia during cooler, drier climatic conditions is well documented, with parallel evidence from disparate sources such as palaeoecology [15], bioclimatic modelling [16], [17], comparative mitochondrial DNA (mtDNA) phylogeographies [18], [19], and multi-locus coalescent analyses [20]. Despite the generation of deeply divergent genetic lineages in vertebrates of Australian Wet Tropics rainforest, refugial isolation has typically failed to generate any corresponding morphological divergence [e.g. 18], [but see 21]. Overall, most vertebrate species appear to have diversified at a larger spatial scale. This is evident in that, in most cases, sister taxa occur in areas (usually rainforest) outside of the Wet Tropics [e.g. 22].

**Figure 1:**
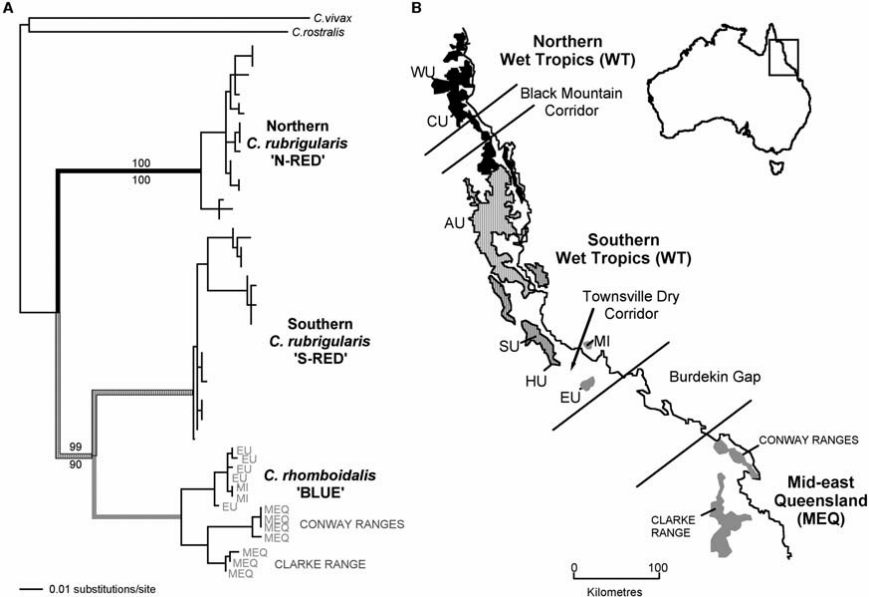
Phylogenetic relationships and spatial distributions of the three lineages of rainbow skinks. A) Phylogenetic relationships among N-RED, S-RED and BLUE according to mitochondrial gene (ND4) maximum likelihood tree [20]. B) The map shows the extent of Wet Tropics and Mid-east Queensland rainforest, and the biogeographical distribution of each of the three Carlia lineages. The shades of lineages on the phylogeny correlate with shades of biogeographic distributions in the map of north-eastern Australia: N-RED in black, S-RED in hashed and BLUE in grey. For female preference trials, three populations from each lineage were collected: N-RED from Windsor Uplands (WU), Carbine Uplands (CU) northern Lamb Uplands (LU); S-RED from northern and southern Atherton Uplands (AU), Spec Uplands (SU); and BLUE from Mt. Elliot (EU), and Finch Hatton in the Clarke Ranges and Brandy Creek in the Conway Ranges in mid-east Queensland (MEQ). Refer to supplementary online material for specific location details. For population demographic analyses, individuals of S-RED were from Spec Uplands (SU) and Halifax Uplands (HU), and BLUE were from Mt. Elliot (EU), and Magnetic Island (MI).

The subjects of the present study are three lineages of rainbow skinks (*Carlia*), restricted to rainforests of mid and north coastal Queensland, Australia. The red-throated rainbow skink, *Carlia rubrigularis* (Ingram & Covacevich 1989) is divided into two genetically distinct lineages, which inhabit Wet Tropics (WT) rainforest, roughly to the north and south of the Black Mountain Corridor, a well-established historical barrier to gene flow [e.g. 22]. These lineages (hereafter referred to as N-RED and S-RED respectively for simplicity) show no morphological divergence, but both lineages vary in body size across ecotones between rainforest and open forest [23]. The third lineage, the blue-throated rainbow skink, *Carlia rhomboidalis* (Peters, 1869) (hereafter referred to as BLUE), is morphologically similar but markedly different in throat colour, a sexually dimorphic character [24]. BLUE inhabits southern outlying Wet Tropics rainforest approximately 35 km to the south of *C. rubrigularis*, across the “Townsville Dry Corridor” and extends to disjunct rainforest patches across the hot and dry “Burdekin Gap” in mid-east Queensland (MEQ; Figure 1). A mtDNA phylogeographic analysis [20] revealed that BLUE is subdivided into two mtDNA clades (WT and MEQ), separated by the Burdekin Gap (see Figure 1).

**Figure 2:**
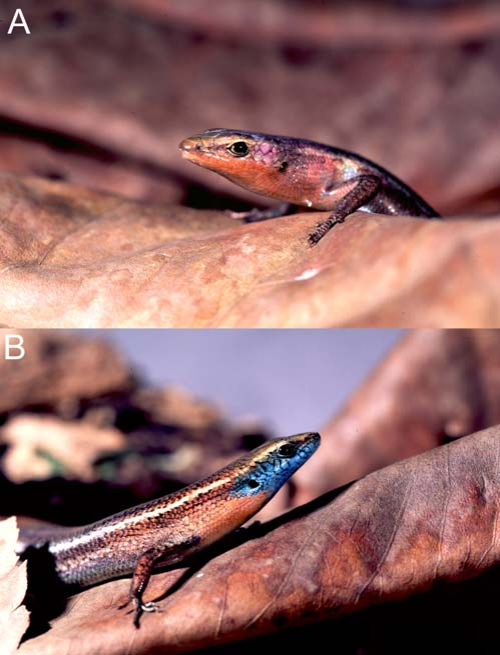
Photographs of typical males showing throat colour difference between the red and blue throated rainbow skinks. A) The red throated rainbow skink, *Carlia rubrigularis* (RED) (N-RED and S-RED are indistinguishable) and B) the blue throated rainbow skink, *C. rhomboidalis* (BLUE). Throat coloration is present in both males and females in the breeding season, but is more vivid in adult males and often absent in females. Photographs: Anthony O'Toole, University of Queensland.

N-RED and S-RED are only readily distinguishable from BLUE by their visually striking throat colour [24], red in *C. rubrigularis* (RED; Figure 2A), and blue with red coloration posterior to the blue in *C. rhomboidalis* (BLUE; Figure 2B). Throat coloration is only produced in the reproductive season and is more vivid in adult males. Behavioural studies in congeneric species, *C. jarnoldae* [25] and *C. rostralis* [26], suggest throat colour, together with chemosensory cues, may be important in mate recognition. The fact that closely related species are often only distinguishable by differences in secondary sexual characters, suggests a role for sexual selection in their divergence [27].

MtDNA phylogeography and multi-locus coalescent analyses [20] suggest that southern S-RED and BLUE diverged more recently than N-RED and S-RED. Multi-locus coalescent analyses estimated that since divergence there has been a low level of lineage-wide introgression between N-RED and S-RED during periods of contact (such as the present), but effectively none (at this lineage-wide scale) between S-RED and BLUE [20]. Morphologically conservative lineages of RED are currently in secondary contact in the central Wet Tropics (Figure 1B). A molecular study of this narrow hybrid zone provided evidence for substantial post-mating isolation (with effective selection against heterozygotes between 22 and 70%, based on patterns of genetic disequilibria) but no evidence for pre-mating isolation [28]. This study provided valuable insight into current interactions between N-RED and S-RED, but a comparable study between S-RED and BLUE is not possible because under current climatic conditions they are separated by approximately 35 km of unsuitably dry habitat (the Townsville Dry Corridor). However paleodistribution modelling (e.g. Moussalli & Dolman, unpublished models) [and see 16] suggest that rainforest was continuous across this region during past cooler, wetter climatic conditions (∼7500-6000 YBP), possibly allowing for interactions between S-RED and BLUE since their initial divergence.

In this study a multidisciplinary approach is used to examine two predictions regarding the contrasting divergences of N-RED and S-RED, and S-RED and BLUE. Firstly, female preference trials are used to test the prediction that assortative female preference is associated with more recent divergence of S-RED and BLUE, but not with the deeper divergence of N-RED and S-RED. Secondly, that upon secondary contact, morphological differentiation (other than throat colour) is maintained between S-RED and BLUE, whereas N-RED and S-RED remain morphogically indistinguishable. An assumption of the second prediction is that S-RED and BLUE have experienced periods of secondary contact, upon which morphological differentiation has been maintained. To test this assumption, multi-locus coalescent analyses are used to estimate post-divergence gene flow between proximally located S-RED and BLUE populations.

## Results

### Female Preference Trials

Focal females spent significantly more time with stimulus males than in a neutral zone (all female types *P*<0.001). Females behaved in a range of ways: from constantly wandering back and forward between stimulus males, to spending the majority of time with one stimulus male. Data are presented as proportion of females that spent greater than 50% of time with males of the same lineage rather than the divergent lineage, and the mean percentage (±SE) of time spent with males of the same lineage (Table 1).

**Table 1:** Results of female preference trials.

Divergence	Lineage of Focal Female	Population of Focal Female	Proportion of females that spent>50% time with males of same lineage		Binomial test P value	Proportion of time females spent with same lineage
N-RED/S-RED	N-RED	LU	3/10	14/30	0.8556	0.421±0.064
		CU	5/10			0.521±0.056
		WU	6/10			0.562±0.055
	S-RED	AU (Nth)	4/10	15/30	1.1444	0.504±0.078
		AU (Sth)	6/10			0.578±0.104
		SU	5/10			0.547±0.095
S-RED/BLUE	S-RED	SU	6/10	16/30	0.8556	0.593±0.096
		AU (Sth)	5/10			0.528±0.084
		AU (Nth)	5/10			0.49±0.036
	BLUE	EU	9/10	23/30	0.0052	0.619±0.062
		Conway Ranges	9/10			0.606±0.073
		Clarke Range	5/10			0.492±0.092

For the N-RED/S-RED divergence, 50% of N-RED females (15 of 30) spent more than 50% of time associated with N-RED males, and 46.67% of S-RED females (14 of 30) spent more than 50% of time associated with S-RED males. For the S-RED/BLUE divergence, 53.33% of S-RED females (16/30) spent more than 50% of time associated with S-RED females. Thus, in all three cases, the proportion of females preferring males of the same lineage did not differ significantly from 50%. In contrast 76.67% of BLUE females (23/30) spent more than 50% of time associated with BLUE males (Binomial test, Bonferroni adjusted α = 0.01, *P* = 0.0052); (90% of females (9/10) from each of Conway Ranges and Mt. Elliot (EU) spent more than 50% of time associated with BLUE males, while only 50% of females (5/10) from Clarke Ranges spent more than 50% of time associated with BLUE males). The proportions of BLUE females from EU and Conway Ranges that preferred BLUE males were significantly greater than the proportions of assortative preference in all other populations of females (t-test, *t* = −13.12, d.f. = 9, Bonferroni adjusted α = 0.01, *P*<0.0002). The times BLUE females from EU and Conway Ranges spent with BLUE males were significantly greater than 50% (One sample t-test, *t* = 2.40. d.f. = 19, α = 0.05, *P* = 0.0266).

### Historical Demography and Gene Flow

Post-divergence introgression was investigated at a local scale between proximal populations of S-RED (SU and HU; Figure 1) and BLUE (EU and MI; Figure 1) by using a coalescent-based method (IM) [29] to estimate population demographic parameters associated with divergence. Inferred highest probability of population migration rates (represented as 2 Nm) between adjacent populations of S-RED and BLUE were effectively zero from S-RED to BLUE (90% highest posterior density, HPD: 0–0.34), but positive (albeit low) from BLUE into S-RED (0.29, 90% HPD: 0.05–0.61). At a gene flow estimate of zero from BLUE into S-RED, the posterior probability is effectively zero (*P* = 0.00007). Therefore zero gene-flow in this direction can be rejected. In contrast, the highest posterior density of gene flow from S-RED to BLUE was in the smallest interval measured, and the likelihood surface was steep and strictly decreasing. Although, a low level of gene flow cannot be ruled out, gene flow from S-RED to BLUE is interpreted to be zero [30], [31]. As gene flow was detected in the direction from BLUE into S-RED, the number and timing of migration events was investigated for this parameter [31]. All seven loci showed evidence for migration. The inferred number of migration events varied across loci, with modes ranging from 1 to 4 (average = 2.7). In terms of the timing of migration, all loci showed low probability (very close to zero) for gene flow at the current time (*t* = 0) and 6 of 7 loci had peak posterior distribution estimates at a time between the timing of divergence and the current time. The inferred timing of migration for the remaining locus was around the time of divergence.

### Morphological Divergence

**Figure 3:**
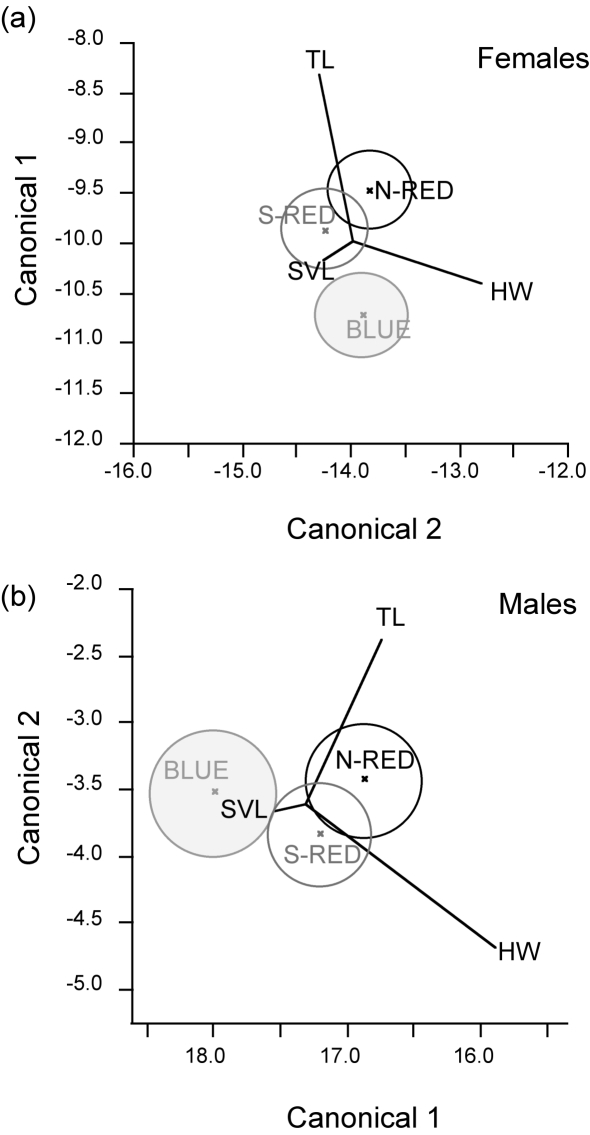
Morphological differentiation between N-RED, S-RED and BLUE. Canonical centroid plots from discriminant analyses for a) females; and b) males. All parameter variables: log snout vent length (SVL), head width (HW), hind limb tibia length (TL) are represented as vectors, with the length of each vector indicating its ability to separate the groups and its direction assists in the interpretation of these differences. Circles represent the 95% confidence intervals around the lineage's centroid.

There were significant levels of morphological differentiation among the three lineages for both females and males (*P*<0.0001, *P*<0.0005, respectively). Focusing in on each divergence event, there was significant differentiation between S-RED and BLUE in both females and males (*P* = 0.0051, *P* = 0.0036, respectively), with BLUE females having shorter hind limb tibial length, and BLUE males having narrower heads than their S-RED counterparts. Univariate trait values (prior to being rendered size-free) also show this trend (female hind limb: N-RED = 7.81±0.07 mm, S-RED = 7.82±0.07 mm, BLUE = 7.63±0.07; male head width: N-RED = 8.06±0.09 mm, S-RED = 8.01±0.08 mm, BLUE = 7.84±0.09). In contrast, although statistical significance was approached, differentiation between N-RED and S-RED did not reach statistical significance in either sex (females *P* = 0.0504; males *P* = 0.0905; α = 0.05). Multivariate differentiation between lineages for each divergence is illustrated with canonical centroid plots from discriminant function analysis (DFA; Figure 3). Testing for homogeneity of multivariate morphology within lineages, BLUE males had significant inter-population variation, with BLUE males from Conway Ranges having significantly greater snout vent length, *P*<0.0001). No other statistically significant differentiation was found within lineages.

## Discussion

Genetic divergences due to refugial isolation of vertebrates on either side of the Black Mountain Corridor in Australia's Wet Tropics rainforest typically have no corresponding morphological differentiation. Instead, sister taxa tend to occur in areas (usually rainforest) outside the Wet Tropics. This is true of *C. rubrigularis* (RED) in the Wet Tropics and its closest relative, *C. rhomboidalis* (BLUE) which inhabits disjunct rainforest to the south of the Wet Tropics. N-RED and S-RED are morphologically indistinguishable, while BLUE has diverged more recently and is only readily distinguishable by a sexually dimorphic trait, throat colour. In order to compare the evolutionary processes leading to different responses to refugial isolation, laboratory-based female preference trials were conducted between N-RED and S-RED, and S-RED and BLUE. Assortative female preference was predicted to have evolved in association with more recent divergence of S-RED and BLUE, but not with the deeper divergence of N-RED and S-RED. These predictions were partly supported by the female preference trials. Between S-RED and BLUE, a significant proportion of BLUE females preferred to associate with BLUE males rather than S-RED males, whereas S-RED females showed no preference. The proportion of BLUE that showed assortative female preference was significantly greater than the proportion of S-RED, suggesting evidence for asymmetric assortative female preference between these lineages. In contrast there was no evidence for assortative female preference between N-RED and S-RED. The lack of assortative female preference found between N-RED and S-RED is consistent with inference from a molecular study of the hybrid zone of this pair of lineages [28], which found evidence for substantial post-mating isolation but no pre-mating isolation.

Before testing the second prediction that S-RED and BLUE have maintained morphological differentiation despite periods of secondary contact, it was necessary to confirm that S-RED and BLUE have indeed experienced periods of secondary contact (e.g. potentially during past cooler, wetter climatic conditions (∼7500-6000 YBP: Moussalli & Dolman, unpublished models). Estimates of post-divergence gene flow revealed a positive, but low level of introgression from BLUE to S-RED, but effectively no introgression in the opposite direction. Coalescent estimates of a positive level of gene flow (albeit low) from proximal populations of BLUE into proximal populations of RED differs from the lack of gene flow inferred from lineage-wide comparisons [20]. This difference is interpreted as a local demographic effect between populations currently isolated from their larger conspecific populations by natural habitat barriers, as opposed to a broad lineage-wide introgression. The inferred number of migration events varied across loci from 1 to 4 (average = 2.7). The timing of migration confirmed the lack of gene flow at the current time (35 km of dry habitat separates these lineages at present), and 6 of 7 loci provided support for gene flow between the timing of divergence and the current time. Thus, there is evidence to suggest that S-RED and BLUE have had periods of secondary contact, upon which any morphological differentiation must be maintained.

In BLUE, this morphological distinctiveness is not only manifested in the sexually dimorphic trait, throat colour, but morphemetric analyses suggest that it is also evident in other morphometric traits. BLUE males have diverged in head width, being narrower than RED males, while BLUE females have shorter hind limb tibia length than their RED relatives. In contrast, morphometric analyses in RED confirmed the lack of morphological divergence in the Wet Tropics: N-red and S-red lineages were morphologically indistinguishable for both sexes. It should be noted, that these morphological measures are sensitive to variation in age structure or growth rates as lizards typically begin reproducing before they attain maximum body size [e.g. 32]. Therefore these results rely on the assumption that age structure was sampled consistently across the three lineages. In conjunction with the lack of assortative female preference between N-RED and S-RED, no morphometric parameters that could act as visual cues for assortative female preference were found. The morphological differentiation between S-RED and BLUE could be a result of sexual selection, with the potential proximal visual cues for assortative preference in BLUE females being male head width, as well as potentially throat colour. Olfactory cues were not directly studied here, but could possibly play a role [see 25], [26]. It must be stressed that the aim of this study was to test for evidence of assortative female preference among the three lineages of skinks; it has not demonstrated a role for throat colour in female preference. Further experiments involving the manipulation of male throat colour are needed to directly examine the role of this sexually dimorphic trait in female preference.

The lack of gene flow at a lineage-wide scale between S-RED and BLUE, compared to a positive level of gene flow between N-RED and S-RED (albeit low) [20] may suggest that barriers to gene flow have been more effective between S-RED and BLUE (notwithstanding potential temporal differences in habitat connectivity). Likewise, greater morphometric differentiation between S-RED and BLUE than between N-RED and S-RED concords with the hypothesis that genetic drift (and/or selection) between S-RED and BLUE have been less affected by the homogenising effects of gene flow. Taken together, relative estimates of lineage-wide gene flow [20], greater morphological differentiation, evidence for periods of secondary contact, and assortative female preference (albeit potentially biased) between S-RED and BLUE, are consistent with the expectation that pre-mating isolation is a more effective barrier to gene flow than post-mating isolation [see also 21].

The variation in assortative female preference evident in BLUE requires further exploration. One possible explanation for the lack of preference in the Clarke Range population is that female sexual receptivity might be delayed in this population as it is the most southerly located population. More speculatively, stronger female preference in the more adjacent populations to RED is a pattern expected with reinforcement. Under the reinforcement model, pre-mating isolation evolves in populations at risk of meeting heterospecific mates in order to avoid producing offspring of reduced fitness [4].

The pattern of asymmetric assortative female preference between S-RED and BLUE is concordant with the direction of asymmetric gene flow estimated from the multi-locus coalescent analysis if dispersal is male biased. That is, if dispersal is male biased, the more discriminating BLUE females would prevent gene flow from S-RED males into the BLUE population, while the less discriminating S-RED females would allow gene flow from BLUE males into the S-RED population. Dispersal has been shown to be male-biased in another Wet Tropics rainforest skink, *Gnypetoscincus queenslandiae* [33] and in reptiles more generally [34]–[36].

It is relatively common for hybridisation of species or populations to be characterised by stronger isolation in one direction than the other [37]. High profile examples include *Drosophila* [38], [39] and salamanders [40]. It has been argued that such asymmetries reflect the direction of evolution, and that females from ancestral populations will discriminate against derived males [38]. According to [38] the asymmetric hybridisation between species of Hawaiian *Drosophila* resulted from loss of courtship display behaviour among the more derived lineages due to small population size-induced drift and founder events. Thus females from the daughter population accept male courtship displays from the ancestral population, but the ancestral females are not attracted to derived males who have lost the courtship displays. The opposite direction of asymmetry is predicted if traits were added rather than lost in more derived lineages. In the current study, maximum likelihood reconstruction of the ancestral state of throat colour suggests that blue colouration is the derived state (Dolman and Stuart-Fox, In Review ) and BLUE, the most recently divergent lineage, is more discriminating than S-RED. Therefore, in this case, it follows that BLUE females discriminate against the ancestral RED males who lack the novel (and presumably attractive) blue coloration or other recently derived cue for mate selection.

Past studies have proposed that despite long-term isolation, a lack of morphological divergence between northern and southern Wet Tropics lineages stems from a lack of divergent selection [e.g. 23]. The present study provides some support for this proposal. That N-RED and S-RED are deeply divergent, have evolved substantial post-mating isolation, yet lack morphological differentiation and pre-mating isolation, suggest genetic drift has played a major role in the divergence of these lineages. In contrast, this study is suggestive of assortative female preference (albeit potentially biased) between S-RED and BLUE. Several scenarios of sexual selection together with genetic drift could underlie the evolution of assortative preference in BLUE females, such as habitat specific optimization of sexual signals [41] or Fisherian sexual selection [10]. The location of male breeding colour on a ‘concealed’ region of the body, the throat, rather than on body regions more easily visible to predators [42] suggests that sexual selection is likely to be more important in this case than divergent predator-driven natural selection [e.g. 43], [44].

In summary, refugial isolation and genetic drift alone have been sufficient to produce substantial post-mating isolation in a secondary contact zone within the Wet Tropics rainforest of Australia [28]. A lack of pre-mating isolation at this contact zone is associated with a lack of morphological diversification. In contrast, this study suggests that greater morphological differentiation of the more recently diverged skinks from neighbouring rainforest to the south of the Wet Tropics is associated with assortative female preference (albeit potentially biased). Combined evidence for more substantial morphological divergence of BLUE and a lack of lineage-wide introgression between S-RED and BLUE [20], despite periods of secondary contact of neighbouring populations, is consistent with the suggestion that assortative female preference is more effective than post-mating isolation in maintaining morphological differentiation between divergent populations. While this study provides a valuable starting point by indicating greater assortative female preference of BLUE females under experimental conditions which did not allow actual mating, it provides no direct evidence for pre-mating isolation or that mating between S-RED and BLUE will be directionally biased. Follow-up breeding experiments in captivity, allowing for behavioural interactions (for example male-male territorial interactions) and comparisons of actual mating events and resulting reproductive success, are required to fully appreciate the extent and relative effectiveness of pre-mating isolation compared with post-mating isolation among these lineages of skinks.

## Materials and Methods

### Female Preference Trials

Female preference trials were used to obtain evidence for female preference and potential pre-mating isolation [e.g. 45]. Early in October, 120 adult females and 120 adult males were collected from three sampling localities for each of three lineages of *Carlia* (total of 9 sampling localities). See Figure 1B for map and Table 2 for sampling locality details. All sampling localities (hereafter referred to as populations) represent discrete subregions as described by [46] and are reciprocally monophyletic phylogeographic units according to mtDNA [20]. To maximize the likelihood that females were receptive to males, the most heavily gravid females were collected and were not available for the experiment until after parturition. At all times, males and females were housed separately, with up to five skinks per enclosure, and no mixing of skinks from different sampling localities. A previous study on S-RED and N-RED found that between mid September and late October, 75% of RED females are gravid and gestation time is around 41–43 days [47]. Given this information and personal observation of similar proportions of gravid females in skinks captured later in the season (e.g. December to February), it is reasonable to assume that females reproduce more that once per season. Although the timing of female receptivity post-parturition is unknown in these skinks, best attempts were made to maximise the time between parturition and use of a female in the preference trials.

**Table 2:** Sampling localities for female preference trials and morphometric analyses and sample numbers for morphometric analyses.

Lineage	Throat Colour	Site	Map reference (Figure 1)	Latitude	Longitude	Morphology (N)	
						Females	Males
northern *C. rubrigularis* N-RED	red	Mount Windsor	Windsor Uplands (WU)	16° 17′ 23″	145° 5′ 2″	14	10
		Mount Lewis	Carbine Uplands (CU)	16° 32′ 56″	145° 23′ 13″	9	10
		Pandanas Creek	northern Lamb Uplands (LU)	17° 01′ 32″	145° 35′ 56″	15	11
southern *C. rubrigularis* S-RED	red	Gillies Lookout	northern Atherton Uplands (AU)	17° 10′ 19″	145° 41′ 13″	10	8
		Ravenshoe State Forest	southern Atherton Uplands (AU)	17° 46′ 36″	145° 33′ 20″	15	12
		Paluma	Spec Uplands (SU)	19° 00′ 52″	146° 15′ 59″	14	18
*C. rhomboidalis* BLUE	blue, red	Mount Elliot	Elliot Uplands (EU)	19° 26′ 10″	146° 56′ 51″	19	11
		Brandy Creek	Conway Ranges	20° 20′ 20″	148° 41′ 27″	9	7
		Finch Hatton	Clarke Range	21° 04′ 59″	148° 38′ 14″	10	8

**Figure 4:**
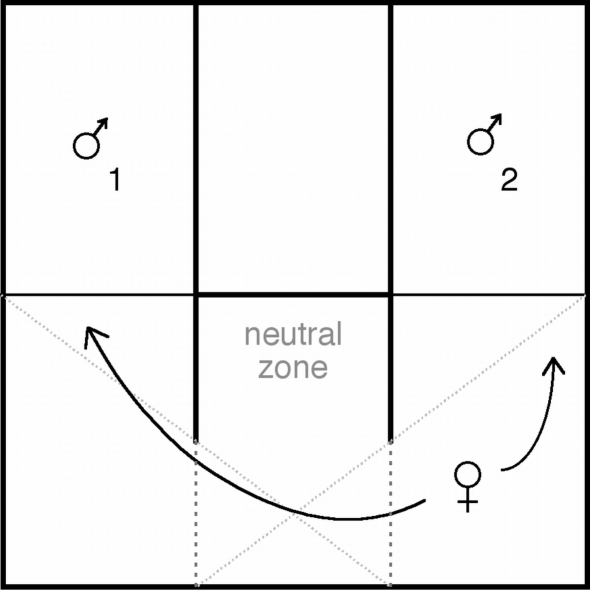
Observation enclosure used for female preference trials. Solid walls extend into the female's quadrant between the two males to form visual barriers so that the female can only see the male in the quadrant she is in (diagonal grey dashed lines mark the line of sight).

A total of 120 female preference trials were conducted (two divergence events, two lineages per divergence event, three populations per lineage, ten females per population). Trials were conducted in an observation enclosure in which the focal female was physically separated from two stimulus males by ultraviolet transparent perspex, replaced by 2 cm of nylon window screen mesh at the base to allow potential olfactory cues to be detected. The female's chamber included a neutral zone, located between the males, where the female could not see either male (see Figure 4). Males were completely isolated from each other and therefore male-male interaction as a causal factor in mate choice was excluded from this experiment. A video recorder was mounted above the enclosure and was used to record the interactions without human interference. However the beginning of each trial was viewed on the video monitor from a distance until the focal female had visited both males (i.e. was presumably aware of the presence of both males) and returned to the neutral zone. Video recording (and data collection) continued for 60 minutes from this point. The two stimulus males were matched for snout vent length (SVL) with the average size difference being 0.3 cm. To control against confounding effects of potential inbreeding avoidance, a female was never offered the choice of a stimulus male from the same sample locality. Trials were conducted in pairs so that within each divergence, two females, one from each alternate lineage, were consecutively given the choice of the same stimulus males. Each female only participated in one trial. To control for unforeseen bias in the female preference observation enclosure, males from a given lineage were alternated between sides 1 and 2 after each paired trial. Videos were reviewed, and times were recorded as the female moved between each zone. The total time the female spent in each zone was calculated for each female preference trial.

Female preference for males from the same lineage versus males of the divergent lineage was tested for the two divergence events. Females in each focal population were offered the choice between a male from the same lineage and a male from the alternative divergent lineage as follows:[Table pone-0003499-t004]

**Table pone-0003499-t004:** 

N-RED/S-RED	30×N-RED females (LU, CU, WU)	N-RED vs. S-RED males
	30×S-RED females (AU-Nth, AU-Sth, SU)	N-RED vs. S-RED males
S-RED/BLUE	30×S-RED females (AU-Nth, AU-Sth, SU)	S-RED vs. BLUE males
	30×BLUE females (EU, Conway, Clarke)	S-RED vs. BLUE males

The male with which the female associated for the most time was determined to be the preferred male. Binomial tests were used to determine whether the proportion of females that preferred males of the same lineage rather than the divergent lineage reached statistical significance. The inbreeding-outbreeding continuum indicates there are no directional predictions for optimal mate preference when comparing populations, therefore all statistical tests of the female preference data are two-tailed. In order to discern relative strength of female preference in populations showing assortative female preference, the percentage of time a female spent associated with a particular male was calculated from the total time she spent associating with both males [e.g. 48].

### Historical Demography and Gene Flow

Historical rainforest contraction has allowed N-RED, S-RED and BLUE to diverge in allopatry. Subsequent habitat expansion has provided opportunity for post-divergence gene flow between N-RED and S-RED, and according to bioclimatic modelling [16] potentially also between S-RED and BLUE. Thus a divergence model that allows for both isolation and migration will improve inference of historical demographic parameters, and allow the detection of post-divergence gene flow. Multi-locus coalescent analyses, implemented in the program IM [29], were previously used to investigate historical demographic parameters in these three lineages of skinks at a broad, lineage-wide scale [20]. Here, I instead assess gene flow between proximal populations of S-RED and BLUE, separated by the Townsville Dry Corridor. The populations compared here are: SU and HU (S-RED) and EU and MI (BLUE; see Figure 1). Each of these populations is currently isolated from conspecific populations by natural habitat barriers.

The demographic parameters estimated using IM are: effective population size of the ancestral population and two daughter populations post-divergence (θ_A_, θ_1_, θ_2_), the directional migration rate between divergent populations (*m*_1_ and *m*_2_; i.e. from population 1 into population 2 and from population 2 into population 1) and the time since divergence (*t*). All parameters are scaled by neutral mutation rate. Post-divergence migration (in each direction), *m* = m/µ is converted to population migration rate (2 Nm) using highest posterior density (HPD) estimates of θ (2 Nm = θ×*m*/2; Hey and Nielsen, 2004).

A total of 17 individuals were sampled from proximal populations of S-RED and BLUE across the Townsville Dry Corridor. Seven previously described nuclear loci: *Aldolase*, *β-globin*, *ets* oncogene, *GAPD*, *MYH*, *Rhodopsin*, and *Sk13-14* [20], [49] were used in the analyses (Genbank accession numbers are provided in Table 3). IM analyses were run with the infinite sites model, with the same recombination-free segments used in [20]. Conditions for the IM analyses were as follows: short runs of 10 million steps were used to refine the parameter limits and heating parameters. Two final runs of eight chains were implemented with geometric heating parameters for 30 million steps, including burn-in of two million steps. Genealogy, branching, demographic parameters and mutation rate mixing, and effective sample sizes were adequate. The two final runs converged on comparable parameter distributions.

**Table 3:** Details of genetic loci used in coalescent-based analyses.

Locus	Locus Description	Genbank Accession #
*Aldolase*	nuclear intron	DQ349410–349443
*β-globin*	nuclear intron	DQ349522–349555
*ets*	nuclear coding	DQ349634–349667
*GAPD*	nuclear intron	DQ349746–349779
*MYH*	nuclear intron	DQ349858–349889
*Rhodopsin*	nuclear intron	DQ349968–350001
*Sk13/14*	Anonymous nuclear	DQ349298–349331

### Morphological Divergence

Snout vent length (SVL), hind limb tibial length (TL) and head width (HW) were measured on live, sexually mature skinks (n = 210) from three sampling localities for each of the three lineages (see Figure 1B, Table 2). Males and females were analysed separately as they were previously shown to be sexually dimorphic [23]. As HW and TL were both highly correlated with SVL, residuals of each of these parameters were calculated from a linear regression against SVL. ‘Size free’ residuals were then used in the analyses. Residuals of HW and TL, and SVL were tested for deviation from normality with Shapiro-Wilk tests, and Bartlett's tests were used to test for heteroscedacity (both tests α = 0.05). Log transformations were necessary to normalize SVL. Morphological characters were analysed with canonical discriminant analysis and multivariate analysis of variance (MANOVA) at three hierarchical phylogenetic scales: i) among three lineages; ii) between each of two divergences and iii) within each lineage.
